# Pneumonie, Zweiklappenendokarditis und Lungenarterienembolie bei einem 26-jährigen Patienten

**DOI:** 10.1007/s00108-021-01228-1

**Published:** 2021-12-22

**Authors:** Lisa Hasselbach, Maximilian Dübbers

**Affiliations:** Universitätsklinik für Innere Medizin, Klinikum Oldenburg AöR, Rahel-Straus-Str. 10, 26133 Oldenburg, Deutschland

**Keywords:** *Coxiella burnetii*, Q‑Fieber, Herzklappenersatz, Levofloxacin, Doxycyclin, *Coxiella burnetii*, Q fever, Heart valve prosthesis, Levofloxacin, Doxycycline

## Abstract

Ein 26-jähriger Patient zeigte eine linksseitige Pneumonie, eine Zweiklappenendokarditis und eine Lungenarterienembolie. Im Jahr 2004 war er bei angeborener Aortenklappenstenose mittels Ross-Operation versorgt worden. Es zeigte sich ein Anstieg der Titer für *Coxiella burnetii*, den Erreger des Q‑Fiebers. Unter antibiotischer Therapie mit Levofloxacin und Doxycyclin kam es zu einer Besserung der Symptomatik und einem Rückgang der Entzündungsparameter. Die Therapie der Endokarditis war erfolgreich.

## Abstract (en)

A 26-year-old male patient presented with left-sided pneumonia, endocarditis of the tricuspid and pulmonary valves and a pulmonary artery embolism. In 2004 a Ross operation was performed because of congenital aortic valve stenosis. There was an elevation of the antibody titer for *Coxiella burnetii*, the pathogen of Q fever. Under antibiotic treatment with levofloxacin and doxycycline there was an improvement of the symptoms and a normalization of the inflammation parameters. The treatment of the endocarditis was successful.

## Anamnese

Ein 26-jähriger Patient stellte sich über die Notaufnahme mit Hämoptysen, linksthorakalen atemabhängigen Schmerzen und Dyspnoe vor. Seit einigen Monaten hätten eine verminderte körperliche Belastbarkeit und vermehrter Nachtschweiß bestanden.

Als einzige Vorerkrankung ist eine angeborene valvuläre Aortenklappenstenose aufzuführen, die 2004 mittels Ross-Operation versorgt wurde. Dabei wurde die patienteneigene Pulmonalklappe auf die Aortenklappenposition versetzt und die native Pulmonalklappe durch ein Homograft ersetzt.

## Körperliche Untersuchung

Bei der Aufnahme war der Patient tachypnoisch mit eingeschränkter pulsoxymetrisch gemessener Sauerstoffsättigung (S_p_O_2_ <90 % unter Raumluft). In der körperlichen Untersuchung zeigte sich ein 3/6-Systolikum mit Punctum maximum über dem zweiten Interkostalraum links parasternal. Der Untersuchungsbefund der Lunge erbrachte einen sonoren Klopfschall bei vesikulärem Atemgeräusch ohne Rasselgeräusche. Es bestanden keine immunologischen Phänomene wie Osler-Knötchen und auch keine Gefäßphänomene wie periphere (septische) Embolien bzw. Janeway-Läsionen.

## Diagnostik

In der *Laboruntersuchung* zeigten sich die Entzündungsparameter und D‑Dimere erhöht.

**Figure 1:**
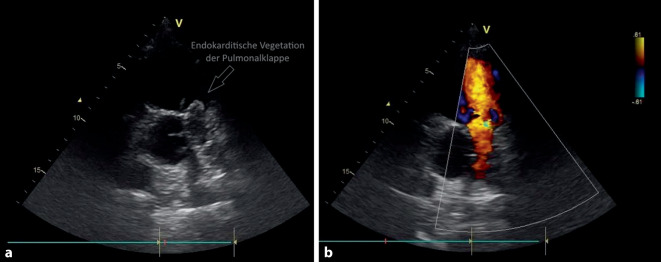

Aufgrund der stattgehabten Ross-Operation wurde noch in der Notaufnahme eine *transthorakale Echokardiographie* durchgeführt, in der eine rechtsventrikuläre und -atriale Dilatation mit reduzierter rechtsventrikulärer Pumpfunktion („tricuspid anular plane systolic excursion“ [TAPSE] 14 mm) dargestellt werden konnte. Das auf Pulmonalklappenposition befindliche Homograft wirkte destruiert und hochgradig insuffizient mit endokarditisch imponierender Vegetation. Auch auf der Trikuspidalklappe ließ sich eine vegetationsverdächtige Struktur nachweisen (Abb. 1). Darüber hinaus zeigte sich der pulmonalarterielle Druck deutlich erhöht (systolischer pulmonalarterieller Druck 60 mm Hg + zentraler Venendruck), sodass eine *Computertomographie des Thorax mit Kontrastmittel* durchgeführt wurde. Hierin sahen wir eine linksseitige Lungenarterienembolie mit einer Lobärpneumonie.

Trotz bestätigter Endokarditis blieben Blutkulturen und Pleurapunktat ohne Erregernachweis. Entsprechend der Leitlinie der European Society of Cardiology (ESC) für Blutkultur-negative infektiöse Endokarditiden erfolgte die *serologische Testung* auf Coxiellen, Bartonellen, Legionellen, Brucellen und Mykoplasmen [1].

Eine *Polymerase-Kettenreaktion* (PCR) für *Coxiella burnetii* war negativ. *Serologisch* zeigte sich ein Anstieg der Immunglobulin-G(IgG)-Antikörper-Titer gegen Phase-1- und Phase-2-Antigene von *C. burnetii*. Die Immunglobulin-M(IgM)-Antikörper-Titer für Phase-1-Antigene waren grenzwertig. Nach 4 Wochen zeigte sich ein weiterer Anstieg der IgG-Antikörper-Titer für Phase-1- und Phase-2-Antigene (Tab. 1).

**Table 1:** 

Antikörper	Referenzbereich	Bei Aufnahme	Kontrolle nach 28 Tagen
*Coxiella burnetii*-IgG-Phase-1-IFT	<1:16	1:64	1:256
*Coxiella burnetii*-IgG-Phase-2-IFT	<1:16	1:64	1:256
*Coxiella burnetii*-IgM-Phase-1-IFT	<1:16	1:16	<1:16
*Coxiella burnetii*-IgM-Phase-2-IFT	<1:16	<1:16	1:16

*IFT* Immunfluoreszenztest, *IgG* Immunglobulin G, *IgM* Immunglobulin M

## Diagnose

-  Endokarditis bei Infektion mit *Coxiella burnetii*

## Therapie und Verlauf

Wir leiteten bei Erstvorstellung unverzüglich eine kalkulierte antibiotische Therapie mit Gentamicin, Vancomycin und Clarithromycin ein. Ein Rückgang der Entzündungsparameter oder der Symptomatik blieb aus. Am fünften Behandlungstag wurde die Gabe von Clarithromycin beendet und Piperacillin/Tazobactam ergänzt.

**Figure 2:**
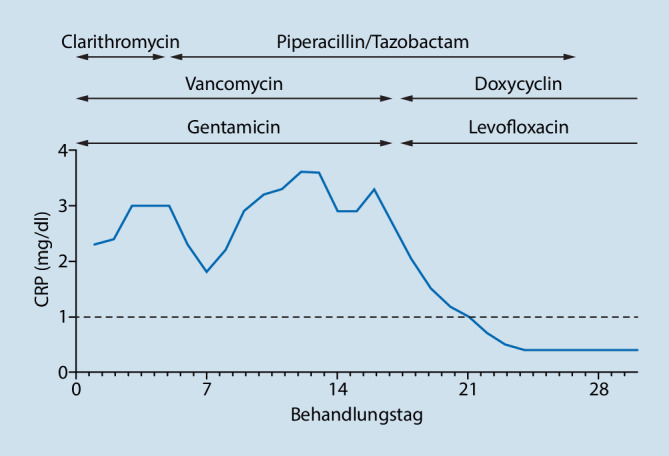

Nach dem Nachweis der Antikörper gegen *C. burnetii* erfolgte die Umstellung der Antibiotika auf Levofloxacin und Doxycyclin. Hierunter zeigte sich die klinische Symptomatik rückläufig, das Infiltrat war im Röntgenbild des Thorax regredient und die Entzündungsparameter normalisierten sich (Abb. 2).

Nach Entlassung wurde der Patient in unserer Ambulanz für Erwachsene mit angeborenen Herzfehlern weiterbetreut. Vier Monate nach Entlassung waren keine endokarditischen Vegetationen mehr nachweisbar. Aufgrund der fortbestehenden hochgradigen Insuffizienz des Pulmonalklappen-Conduit erfolgte etwa 8 Monate nach der Erstvorstellung ein operativer Klappenersatz.

## Diskussion

### *Coxiella burnetii*

*C. burnetii* ist ein intrazelluläres, gramnegatives Bakterium, das in enger Verwandtschaft zu Legionellen steht [2]. Im Jahr 2019 wurden 150 Infektionsfälle aus Deutschland an das Robert Koch-Institut (RKI) gemeldet, aufgrund der unspezifischen Klinik muss von einer hohen Dunkelziffer ausgegangen werden [3]. Die Inkubationszeit beträgt 7–32 Tage [4].

Eine Herzklappenprothese ist ein unabhängiger Risikofaktor für eine Chronifizierung des Q‑Fiebers

Die meisten Infektionen verlaufen asymptomatisch. Man unterscheidet einen akuten Krankheitsverlauf mit Pneumonie, Hepatitis und Fieber von einem sehr seltenen chronischen Krankheitsverlauf, typischerweise mit einer Endokarditis. In seltenen Fällen kann es im Rahmen der Akutinfektion zu einer Endokarditis kommen [2, 5]. Eine Herzklappenprothese ist ein unabhängiger Risikofaktor für eine Chronifizierung des Q‑Fiebers [6].

### Q-Fieber: Diagnostik und Therapie

Zur Diagnostik des Q‑Fiebers eignet sich die serologische Untersuchung mit Nachweis von IgM- und IgG-Antikörpern gegen *C. burnetii*. Ein Antigen-Shift von Phase-1- zu Phase-2-Antikörpern gilt als Anzeichen für eine weiter zurückliegende Infektion. Coxiellen-DNA ist zu Beginn der akuten Infektionsphase mittels PCR nachweisbar [7].

Bei einer durch *C. burnetii* hervorgerufenen Endokarditis als Zeichen einer Chronifizierung sollte die antibiotische Therapie für 18 Monate (Nativklappen) oder für 24 Monate (Klappenprothesen) fortgeführt werden. International wird meist eine Therapie mit Doxycyclin und Hydroxychloroquin favorisiert [8]. Wir haben uns an der Empfehlung des RKI orientiert und Doxycyclin und Levofloxacin verabreicht.

### Unser Patient

Der Patient zeigte eine Pneumonie, eine Zweiklappenendokarditis und eine Lungenarterienembolie ohne Keimnachweis trotz mehrfacher Entnahme von Blutkulturen. Anfänglich bestanden Zweifel an der Validität der Diagnose, denn die PCR-Diagnose erbrachte keinen DNA-Nachweis und zur Diagnosestellung würde ein IgG-Titer von mindestens 1:200 erwartet werden. Alternativ bestätigt jedoch auch ein 4‑facher Anstieg des IgG-Antikörper-Titers innerhalb von 3–6 Wochen die Diagnose [7, 9]. Im vorgestellten Fall zeigte sich dieser nach 28 Tagen.

Passend zur serologischen Diagnostik sahen wir erst nach Umstellung der antibiotischen Therapie mit Abdeckung der intrazellulären Erregerspezies eine klinische Beschwerdebesserung sowie einen Rückgang der Infektparameter und der endokarditischen Auflagerungen. Entsprechend der ESC-Empfehlung erfolgte eine serologische Testung auf weitere relevante intrazelluläre Erreger (Chlamydien, Rickettsien, Mykobakterien, Bartonellen), die von uns als Differenzialdiagnosen in Betracht gezogen wurden. Für die genannten Erreger gelang kein Nachweis eines erhöhten Titers, sodass sich die Verdachtsdiagnose des Q‑Fiebers weiter festigte.

Bei nur etwa 1 % der Infektionen mit *C. burnetii* kommt es zu einer Chronifizierung, meist im Sinne einer Endokarditis. Die Chronifizierung tritt üblicherweise bei Patienten mit vorbestehender Herzklappenerkrankung oder einer Immunsuppression auf [3]. Bei den Besuchen im landwirtschaftlichen Betrieb der Eltern seiner Lebensgefährtin bestand kein direkter Tierkontakt, jedoch wird *C. burnetii* auch über kothaltige Stäube übertragen. Der Patient gab an, die Stallanlagen betreten zu haben, sodass wir diesen Infektionsweg für am wahrscheinlichsten halten [10]. Zur Diskussion stand auch eine selten berichtete sexuelle Übertragung des Erregers [11], diese konnte durch einen negativen Antikörpertiter für *C. burnetii* bei der Partnerin ausgeschlossen werden.

Diskussionswürdig ist die zeitliche Einordnung des Krankheitsgeschehens. Es wäre denkbar, dass die Pneumonie den Ort der Primärinfektion mit *C. burnetii* anzeigt und Ausgangspunkt einer Bakteriämie und damit Auslöser der Endokarditis war und dass die Krankenhausaufnahme am Beginn der Chronifizierung erfolgte, sodass sowohl die Pneumonie als Zeichen der akuten Erkrankung als auch die Endokarditis als Zeichen der chronischen Phase sichtbar war. Eine alternative Erklärung wäre, dass der Patient primär eine Endokarditis entwickelte und dann im Rahmen der Lungenarterienembolie eine Infarktpneumonie auftrat. Für Letzteres spricht, dass der ansonsten immunkompetente Patient in Form seines Homografts auf der Pulmonalklappenposition eine Prädilektionsstelle besaß. Von dort aus kam es zum subklinischen chronischen Krankheitsprozess mit plötzlicher Akzeleration der Beschwerden ab der septischen Embolie.

## Fazit für die Praxis

- Bei Blutkultur-negativer Endokarditis sollte eine Infektion mit *Coxiella burnetii* ausgeschlossen werden.
- Im Falle einer Klappenprothese erfordert das Q‑Fieber eine antibiotische Therapie über 24 Monate.
